# Editorial: What’s Love Got to Do With It: The Evolution of Monogamy

**DOI:** 10.3389/fevo.2020.00110

**Published:** 2020-04-28

**Authors:** Nancy G. Solomon, Alexander G. Ophir

**Affiliations:** 1 Department of Biology, Miami University, Oxford, OH, United States; 2 Department of Psychology, Cornell University, Ithaca, NY, United States

**Keywords:** social monogamy, genetic monogamy, infidelity, biparental care, spatio-temporal relationship, pair bond

Monogamy and pair-bonding are central to the human experience in the majority of cultures worldwide (Schacht and Kramer), which might explain the long-running fascination scientists have for understanding monogamy within mammals and across other taxa. The inherent interest in monogamy in western cultures, in part, may be a result of anthropomorphism and a belief that who we mate with defines us. Nevertheless, monogamy captivates the human mind and has been the subject matter in art, religion and literature for centuries. It is a topic that has brought together researchers from diverse backgrounds including anthropology, behavioral ecology, psychology, psychiatry, pediatrics, neurobiology, endocrinology, and molecular biology.

There is still much we do not understand about monogamy. A collective, systematic, and concerted effort toward answering questions surrounding the meaning of monogamy is overdue. This Research Topic aimed to bring experts, from a variety of disciplines and conceptual approaches, together to showcase our current understanding of monogamy. This issue is composed of articles focusing on the specific and general aspects of monogamy within a variety of species, and taking empirical, methodological, conceptual, or theoretical approaches to provide a deeper and more complete understanding of aspects of behavior that comprise monogamy, its evolution, and its meaning.

The term “monogamy” can be used in very different contexts or ways, emphasizing the need to carefully delineate or define terminology. This is a critical concern because not only can there be confusion between different forms of monogamy (e.g., “social” and “genetic” monogamy), but also about the particular behaviors that should be included within the concept of monogamy. Thus, consistent and clearly defined terminology is crucial, especially when conducting comparative analyses (Huck et al.; Kappeler and von Schaik, 2002). Early studies of monogamy often assumed that animals with a high degree of spatio-temporal overlap mated exclusively with each other (Wittenberger and Tilson, 1980). Since the advent of molecular techniques enabling parentage determination, it has become clear that exclusive mating with a social partner (i.e., genetic monogamy) is much rarer than social partnerships in which mating outside the pair occurs (e.g., eastern bluebirds, *Silia silis,* Gowaty and Karlin, 1984, indigo buntings, *Passerina cyanea,* Westneat, 1987; fat-tailed dwarf lemur, *Cheirogalaus medius,* Fietz et al., 2000). Thus, the propensity for two opposite sexed individuals to live together need not relate to an exclusive mating relationship, in itself requiring a reevaluation of the common understanding of monogamy.

Social monogamy can be defined in terms of spatial overlap of one adult male and one adult female that live as a pair. Advances in methods to study such behavior in nature, particularly among cryptic subterranean species (like rodents), provide accurate and more refined determinations of behavior that can significantly improve assessment of the behaviors that define monogamy. For example, Sabol et al. demonstrated that incorporating automated radio frequency identification ‏tracking (RFID) and social network analysis can provide new opportunities to measure and operationalize monogamous behavior beyond classic laboratory tests of partner preferences (Williams et al., 1992; Donaldson et al., 2010) and assessment of home ranges and spatial overlap using radiotelemetry in the field (Solomon and Jacquot, 2002; Ophir et al., 2008; Lambert, 2018).

Although the spatio-temporal relationship between mating partners provides one important way to define socially monogamous relationships (Emlen and Oring, 1977; Ophir, 2017), other conceptualizations of monogamy focus more heavily on the suites of behaviors that contribute to delineating this social system. Relying on behavioral characteristics to define monogamy (i.e., social association, formation of an attachment, mating pattern, biparental care of offspring, and selective aggression toward same-sex conspecifics) can lead to different views of monogamy because not all the behaviors that contribute to various conceptualizations of monogamy are consistently found in every socially monogamous species. For example, some of the early reviews of monogamy (Kleiman, 1977; Wittenberger and Tilson, 1980) included biparental care as a key feature of monogamy, but we now know that biparental care is not found in all monogamous animals (Brotherton and Rhodes, 1996; Whiteman and Côté, 2004, Table 2; Lambert et al., this issue Supplementary Data sheet 1). Even when biparental care is common (e.g., in avian monogamy, Mock and Fujioka, 1990), the contributions of parents are not necessarily equal and may not be constant over time. Rogers et al. examines the pattern of biparental care seen across multiple litters and finds that the mother and father appear to compensate for the time spent in parental care by the partner. Similarly, Schacht et al. investigate human paternal investment within a population of Maya living in Mexico in response to changes in socioecological factors. The authors investigate how changes in socioecological factors such as the introduction of mechanized farming, and their potential to provide stability in offspring social environments might compensate for a partner’s waning parental effort or create opportunities for increased effort. For instance, mechanized farming among the Maya increases efficiency in providing subsistence, mating partnerships, and bi-parental care.

Another issue that can confound our understanding of monogamy is the acknowledgment that individuals within a population typically described as monogamous vary. Indeed, any mating system is best considered to be a collection of individual reproductive decisions, which are shaped by many internal and external factors. For example, many species that are considered monogamous contain individuals that engage in alternative mating tactics. Typically, alternative tactics within mating systems tend to take the form of (socially) monogamous residents that maintain territory, site, or nest fidelity with an opposite-sex partner, or the form of non-monogamous (often promiscuous) wandering or roaming individuals that maintain more than one nest and/or lack the site fidelity of the former. How evolution has maintained the variation in tactics is an important question that strikes at the heart of this phenomenon (Dawkins, 1980; Koprowski, 1993; Taborsky et al., 2008). In this issue, Shuster et al. used data from a 3-year study in two geographically separate populations of prairie voles to investigate the average fitness obtained by males and females that exhibited alternative reproductive tactics. They showed that a form of balancing selection could maintain both behavioral phenotypes in the populations. Adopting a socially monogamous resident tactic does not preclude the motivation (or reproductive advantages that presumably support it) to pursue extra-pair matings. So, although the tactics are distinct from each other, an individual can switch from one tactic to another depending on which tactic results in higher fitness under particular environmental conditions. In another paper in this special topic, Rice et al. use optimal performance modeling to predict when it would be better for males that are pair-bonded to stay and guard their mates vs. seek extra-pair copulations in a population where males display alternative mating tactics. The latter would come with the cost of losing matings with their female partner to conspecific males in the population. Together, studies such as these highlight the profound individual variation between and within tactics that exists within “monogamy” and point to external factors (social or ecological) that help shape the evolution of reproductive decision-making.

There are still many unanswered questions that remain about how socially monogamous animals respond to infidelity in their partner. A number of papers in this issue explore different aspects involved in the potential for loss of genetic monogamy in socially monogamous individuals. Pultorak et al. investigates vocalizations among individuals during the formation of a pair-bond and during an “infidelity challenge,” where the male and female were housed for 1 week with an unfamiliar opposite-sex conspecific. Maninger et al. used functional imaging to investigate the changes in cerebral glucose metabolism, and an array of hormones taken from brain and blood after a pair-bonded male titi monkey observed his partner in close proximity with a rival male. Maninger et al. provocatively suggest that the experience induced a neural and physiological response indicative of “jealousy” and that such responses preserve pair bonds.

In recent years, a focus on proximate studies like the latter one, and recognition for the tremendous value toward providing a complete understanding of behavior (sensu Tinbergen, 1963), has increased. Studies about the neurogenetic mechanisms underlying characteristics of monogamy, particularly the formation of pair bonds, have received considerable attention (Young and Wang, 2004; Klatt and Goodson, 2013; Fischer et al., 2019) and have been major contributors toward this wave of interest in proximate behavioral mechanisms. Nevertheless, the mechanistic underpinnings of monogamy are complex, and important outstanding questions remain about the relative importance and interrelationship among neurogenetic factors. In a remarkably comprehensive review, Carter and Perkeybile outline many of the behavioral, hormonal, neural and genetic/epigenetic mechanisms that contribute to mating behavior, the formation and maintenance of the pair bond, the emotion humans call “love,” and the gulf between them. Similarly, Carp et al. focus on the role of dopamine in the length of time animals have been paired to assess the function of this neurotransmitter in the strength of bonds. Together, these papers provide insight into the deep evolutionary roots of shared‏ mechanisms that govern short-term or long-term monogamous relationships, while also demonstrating that there is no one single neurochemical recipe for social monogamy. Finally, ontogenetic effects on monogamy have received comparably little attention compared to Tinbergen’s other levels of analysis, though such studies do exist. For example, rodent studies have clearly demonstrated that early-life experience can alter the propensity for monogamy (Bales et al., 2007; Ahern and Young, 2009; Prounis et al., 2015), and the neurochemical substrates that contribute to it (Hiura and Ophir, 2018; Hiura et al., 2018; Kelly et al., 2018; Prounis et al., 2018). Similarly, Al-Naimi et al. examines the effects of social and environmental disruptions in the life of young animals, and discuss how these may affect the tendency of males to behave monogamously.

Many studies of monogamy naturally lead to questions that address the adaptive value of this mating system, but the importance of understanding monogamy from the comparative evolutionary, mechanistic, and developmental perspectives (sensu Tinbergen, 1963) cannot be understated. Questions about the evolution of monogamy have been a steadfast area of interest in behavioral ecology and the study of animal behavior. In the 1980–2000s, the costs and benefits of monogamy to males and females received a lot of attention (Wittenberger and Tilson, 1980; van Schaik and Dunbar, 1990; Hames, 1996; Reavis and Barlow, 1998), and numerous hypotheses were proposed and tested in different species. Since then, great advances in phylogenetic reconstruction have paved the way for a better understanding of the evolution of social monogamy, including the causal factors that led to social monogamy and the factors that were consequences of this social/mating system (Dobson et al., 2010; Lukas and Clutton-Brock, 2013). Previous phylogenetic studies on factors influencing genetic monogamy have not reached consistent conclusions for a number of reasons, including differences and limitations in methodology and the species included in analyses (Clutton-Brock and Isvaran, 2006; Cohas and Allaine, 2009; Huck et al., 2014; Dobson et al., 2018). In this issue, Lambert et al. used phylogenetic corrections to examine factors influencing genetic monogamy within socially monogamous mammals and found that there was not one best model that explained the different ways in which genetic monogamy could be assessed. Numerous life history factors such as pair living and paternal care were important contributors to the top models. These, together with some demographic factors such as density or sex ratio, affected some measures of genetic monogamy. This conclusion is consistent with the argument made by Klug, which considered the life-history and ecological conditions that permit the evolution and persistence of monogamy, and advocated for the idea that multiple (interacting) factors influence the display of monogamy. In a more specific example, Macdonald et al., reviewed some of the same functional hypotheses for the evolution and persistence of social and genetic monogamy in wild canids, a mammalian family in which monogamy is common. They proposed the hypothesis that the combination of particular characteristics of canids has led to both monogamy and prosocial cooperation. In another study employing the comparative approach, Kalcounis-Rueppell et al. consider the relationships between the form and function of *Peromyscus* vocalizations with ecological traits, physiological traits, and different mating systems, ranging from polygyny to monogamy, across seven species of mice.

The study of monogamy has accelerated in the past few decades, painting a nuanced, and at times perplexing yet captivating, picture for what monogamy actually is. Such work has only uncovered more exciting questions that merit investigation, and highlight that we are just beginning to understand what monogamy actually is. Love, for example, is—at best—only a small part of this larger phenotypic complex. We hope that the contributed papers contained within this collection will stimulate discussion and promote more research, which in the end, will enhance our understanding of monogamy, and lead to achieving a deeper understating of the mating system often associated with humans and many other species across the animal kingdom.
